# Supplementary material for: Content-rich biological network constructed by mining PubMed abstracts
Source: BMC Bioinformatics. 2004 Oct 8;5:147. doi: 10.1186/1471-2105-5-147 (PMC528731; doi:10.1186/1471-2105-5-147)
Supplement: Additional File 2 — The original results of the above study (non-essential files are deleted to keep the file size under the limit set by BMC bioinformatics). [file 1471-2105-5-147-S2.bz2 › chilibotAdditionalFile2/dip30/202ID8652557E691/html/ERCC2_ERCC5.html]

 


 **ERCC2** and **ERCC5** 
  
Found 28 abstracts in PubMed, retrieved 28.  
 

 What does Google say? 
 PDF only 
| .edu only 

---

**Interactive relationship** (e.g. stimulation, inhibition, etc)

**Neutral relationship**- We suggest that XPG  [ **ERCC5** ]  nuclease acts on the single stranded region created as a result of the combined action of the XPB helicase and XPD  [ **ERCC2** ]  helicase at the DNA damage site.  Ref: 8078765 Nucleic Acids Res, 1994

**Non-interactive relationship** (e.g. studied together, co-existance, homology, etc.)

- Defects in the XPB, XPD  [ **ERCC2** ] , and XPG  [ **ERCC5** ]  genes can result in three different syndromes, xeroderma pigmentosum, Cockayne syndrome, or trichothiodystrophy, depending on the specific mutation involved.  Ref: 9425594 Curr Opin Pediatr, 1997
- However, cells deficient in the other NER genes, XPD  [ **ERCC2** ] , XPB and XPG  [ **ERCC5** ] , all have the same ratio of exchanges to breaks as wild type.  Ref: 12509251 DNA Repair Amst , 2002
- Cell lines defective in other genes involved in NER XPB, XPD  [ **ERCC2** ] , and XPG  [ **ERCC5** ] , together with a mutant defective in nonhomologous end joining XRCC5, showed only mild sensitivity.  Ref: 11027268 Mol Cell Biol, 2000
- In contrast to cells bearing mutations in other components of the nucleotide excision repair apparatus XPB, XPD  [ **ERCC2** ] , XPG  [ **ERCC5** ]  and CSB, cells defective for the ERCC1 XPF structure specific nuclease are highly sensitive to cisplatin.  Ref: 12202770 Nucleic Acids Res, 2002
- On the basis of the estimated initial rates of incision the different XP cells examined in this work can be ranked as follows XP variant much greater than XPH greater than XPH greater than XPD  [ **ERCC2** ]  greater than XPC greater than XPG  [ **ERCC5** ]  greater than XPA.  Ref: 3347209 Mutat Res, 1988
- In each of these cell lines, the genes examined included ERCC1, XPA, XPB, XPD  [ **ERCC2** ] , XPG  [ **ERCC5** ] , CSB and p53.  Ref: 10738106 Cancer Lett, 2000
- We compared the sensitivity of the wild type CHO cell line, AA8, with that of the CHO mutant cell lines UV4 and UV20 ERCC1, UV5  **ERCC2** , UV24 ERCC3, UV41 ERCC4, UV135  **ERCC5** , UV61 ERCC6, and EM9  [ **ERCC2** ]  XRCC1.  Ref: 8765433 Cancer Chemother Pharmacol, 1996
- these can be further subdivided into mutants which are defective in nucleotide excision repair alone UV5  **ERCC2** , UV24 ERCC3, UV135  **ERCC5**  and UV61 ERCC6 compared to those which have an associated defect in the distinct but overlapping pathway for the repair of DNA crosslinks UV20 ERCC1 and UV41 ERCC4.  Ref: 9339946 Radiat Res, 1997
- Loss of heterozygosity LOH analysis, using the microdissected tissues, for the XPA, XPB, XPC, XPD  [ **ERCC2** ] , XPE, XPF, XPG  [ **ERCC5** ]  and the transcription coupled repair factor, Cockayne syndrome B CSB revealed that NER factors were abnormal in 30.0% 3 10 cases of oral squamous cell carcinomas.  Ref: 11496330 Oncol Rep< MedlineTA>Oncol Rep, 1997
- Normally, the ERCC1 excision repair cross complementing and ERCC4 deficient cell lines are most sensitive to DNA cross linking agents, with  **ERCC2** , ERCC3 and  **ERCC5**  deficient cell lines having minimal sensitivity.  Ref: 8053936 Biochem Pharmacol, 1994
- RESULTS Mammalian cell lines XPB, XPD  [ **ERCC2** ] , XPF, XPG  [ **ERCC5** ] , and ERCC1 deficient in the nucleotide excision repair NER gene products show resistance to Et 743.  Ref: 11731295 Chem Biol, 2001
- Xeroderma pigmentosum cells deficient in the NER genes XPG  [ **ERCC5** ] , XPA, XPD  [ **ERCC2** ]  or XPF were resistant to Et743, and sensitivity was restored by complementation with wild type genes.  Ref: 11479630 Nat Med, 2001
- mRNA levels of XPA, XPB, XPC, XPD  [ **ERCC2** ] , XPF, XPG  [ **ERCC5** ] , CSB and ERCC1 in primary lymphocytes from 33 individuals were quantified by dot blots and normalized to beta actin.  Ref: 11056291 Mutat Res, 2000
- Another class of patients with CS symptoms have mutations in the XPB, XPD  [ **ERCC2** ] , or XPG  [ **ERCC5** ]  genes, which result in UV hypersensitivity as well as defective global NER.  Ref: 11443545 Am J Hum Genet, 2001
- Loss of heterozygosity LOH analysis for the XP, XPA, XPB, XPC, XPD  [ **ERCC2** ] , XPE, XPF, XPG  [ **ERCC5** ]  and the transcription coupled repair factor, Cockayne syndrome B CSB revealed that NER factors were abnormal in 62.1 % of ovarian tumors 18 2 6.7% of colon 2 12 and 22.2% lung 2 9 carcinomas.  Ref: 11689286 Cancer Lett, 2001
- We have compared the frequency of polymorphisms in the NER genes, XPD  [ **ERCC2** ] , XPF, XPG  [ **ERCC5** ] , ERCC1.  Ref: 12865926 Br J Cancer, 2003
- Such genes include ERCC1, XPA, XPB, XPD  [ **ERCC2** ] , XPF, and XPG  [ **ERCC5** ] .  Ref: 10891549 Int J Oncol, 2000
